# Designing and Piloting of a Mobile Learning Curriculum for Quality Point-Of-Care Diagnostics Services in Rural Clinics of KwaZulu-Natal, South Africa

**DOI:** 10.3389/frph.2021.728309

**Published:** 2022-02-01

**Authors:** Nkosinothando Chamane, Rowan Thompson, Simon Goldstone, Tivani Phosa Mashamba-Thompson

**Affiliations:** ^1^Department of Public Health Medicine, School of Nursing and Public Health, University of KwaZulu-Natal, Durban, South Africa; ^2^Department of Mathematics, Science and Technology, Stadio School of Education Formerly the Embury Institute for Higher Education, Durban, South Africa; ^3^Department of Digitally Enhanced Learning, Teaching and Assessment, Stadio School of Education Formerly the Embury Institute for Higher Education, Durban, South Africa; ^4^Faculty of Health Sciences, University of Pretoria, Pretoria, South Africa

**Keywords:** point-of-care diagnostics, learning management systems, curriculum delivery, resource limited settings, mobile learning (mLearning)

## Abstract

**Background:**

The use of mobile technology has been reported to help improve access to education for people in remote areas. However, there is limited evidence of its adoption in resource-limited settings. The aim of this study was to utilize stakeholder generated ideas to design and pilot a mobile learning curriculum, with the purpose of facilitating training to improve the quality of point-of-care diagnostics services in KwaZulu-Natal (KZN) rural clinics.

**Methods:**

Nominal Group Technique was employed to enable collaboration with stakeholders in designing and piloting of a POC diagnostics curriculum. Stakeholders were selected from 11 KZN districts to participate in a clinic-based piloting of the curriculum using an online application. The application was designed in collaboration with a teacher training institute in Durban. Moodle was used as an established reliable online learning management system. During piloting, quantitative and qualitative data were generated and analyzed using descriptive statistics and content analysis.

**Findings and Conclusion:**

Guided by the Nominal Group Technique results, five delivery modes for curriculum content through Mobile Learning were generated. An interactive course page was created on the Moodle site, titled: *Quality HIV Point of Care Diagnostics Curriculum Delivery for Nurses in Rural Areas*. The course content consisted of three teaching units, activities, an online quiz and an online survey. An analytic-algorithm built into the online course enabled monitoring of participation and assessment outcomes automatically. At piloting, 64% of the invited representative clinics were able to access the course, with 47% meeting the course completion requirements. All the participants achieved the set pass mark of 75% with an average of 87%. The activity completion report showed that topics presented through images, videos and simple text were accessed more than those presented as attachments of national documents. Despite poor network coverage and limited access to mobile technology, exacerbated by Covid-19 related restrictions, Point of care diagnostics Mobile Learning curriculum was well-received in participating rural clinics. Recommendations relating to course improvement and access, included extending collaboration with specialists in eHealth systems development and with South African cell phone network providers.

## Introduction

Mobile technology has been well-received throughout the world due to its ability to transform education and training (1), making it more accessible to people working in resource-limited settings (2, 3). This technology has the ability to support learning that is contextual and experiential, as well as providing authentic and up-to-date content to health workers in remote areas (1, 3). Mobile learning (mLearning) is defined as the provision of education and training through any portable handheld/wireless device, mobile or smart phone, that can be utilized where necessary (4, 5). However, what counts as mobile technology is changing so rapidly that a definition may be obsolete before this paper reaches the public domain. For example, since the inception of this study, “smart wrist-watch” and “smart glasses” technologies have been launched which may eventually replace devices currently recognized as “smart phones.” For the purposes of this study, mobile technology incorporates the use of portable devices such as smart cell phones, digital tablets and laptop computers as educational tools. The way that the user interacts with the technology, in a mobile sense, requires a connection to the internet which is provided by a telecommunication internet service provider. Over the years, the National Nurses Associations (NNAs) have called for the integration of information technology into nurse education curricula through mLearning to help prepare students for the current practice environment which requires access to large amounts of information necessary to provide evidence-based patient care (6). mLearning has also been successfully incorporated in various nurse education curricula with the aim of enhancing nursing practice education (2).

Lack of training and of resources, and staff shortages leading to challenges when sending personnel away for training, have been highlighted as barriers to the provision of quality point of care (POC) services (7–9). In addition, results of a recent scoping review have demonstrated that there is limited research on clinic-based training approaches for POC diagnostics (10). A previous engagement with POC diagnostics stakeholders from various health districts of KwaZulu Natal (KZN) led to identification of the need for a POC diagnostics curriculum for primary healthcare (PHC) nurses (11). In addition, the lack of continuous professional development (CPD) opportunities was also identified amongst the main challenges to the provision of quality HIV testing (11). This is a concern, especially in this era of gradual phasing out of HIV lay counselors and shifting of HIV/AID testing duties to professional nurses, announced in 2014 (12).

Research has shown that knowledge in the modern world is transformed by the development of revolutionary technologies (5). In the South African context, it is considered vital to develop home-grown technologies to encourage entrepreneurship and reduce unemployment (13). Mobile digital technologies such as this are considered essential to stimulate economic growth and meet the future employment requirements of a growing digitally literate youth population, in an increasingly competitive technology dependent job market. In this study, the aim was to utilize stakeholder-generated delivery approaches to design and to pilot an evidence-based, and context-specific, mLearning curriculum to improve the quality of POC diagnostics services in rural PHC clinics in KZN. It is anticipated that the findings of this study will help inform implementers of CPD programmes for PHC nurses in high disease burdened and resource-limited settings. It is also believed that the study results will help improve health workers' adherence to POC diagnostics quality standards and contribute to strengthening health systems by improving technological literacy in health care professions.

## Materials and Methodology

### Study Design

This study is part of a larger co-creation study, which was aimed at engaging relevant stakeholders in designing a point-of-care (POC) diagnostics curriculum to be delivered through mLearning. In the first component of the larger study a Nominal Group Technique (NGT) (14) was adapted to engage representative stakeholders from 11 districts of KwaZulu-Natal. This was followed by an engagement on the development of an mLearning app for the delivery of a POC diagnostics curriculum for nurses in resource-limited settings. NGT can be defined as a structured small-group discussion to reach consensus. It gathers information through asking individual participants to respond to a question posed by the facilitator through a brainstorm of ideas, followed by the ranking of generated ideas (15, 16). A comprehensive account of the theory part of the NGT methodology employed in this study was presented in this researcher's earlier publication (11). On the second component of the study, the participant' contributions were incorporated in designing a cell phone accessible online application, built in a learning management system to enable trailing of a training curriculum for PHC workers in remote areas of KZN. Details on the participants, sampling, NGT process followed and mLearning application development are provided in the sections below.

### Study Participants

The NGT team consisted of 10 members, namely: three professional nurses, one TB professional nurse, one TB assistant, one HIV/AIDS counselor, two experienced researchers, the primary investigator (as facilitator) and one research assistant. All the participants had previous experience of participating in a nominal group discussion.

### Sampling Strategy

This study is conducted as a follow up to a cross-sectional survey of 100 randomly selected rural PHC clinics of KZN (17), as well as the co-creation study referred to in the study design section (11). The survey was aimed at determining the accessibility, availability and utility of POC diagnostics in rural PHC clinics (17). Following the survey, clinics identified to have the highest availability and usage of POC diagnostics, were selected to participate in this study. Thus, one PHC clinic was included from each of the 11 KZN districts with the highest availability and usage of POC tests. As stated in the first component of this study, a purposive sampling strategy was employed to select representatives from the sampled clinics. A pre-requisite for inclusion in the study was that participants needed to have practical knowledge of PHC-based POC diagnostics.

### NGT Process (Part 2)

The NGT workshop was facilitated by the primary investigator (NC), assisted by a trained research assistant (PS). To open the session the facilitator presented the aim of the session to incorporate stakeholders in the designing of a training approach for the quality delivery of POC diagnostics services based on experiential learning. A similar process to that presented in the previous study (11) was followed. The primary investigator posed the following question for the participants to consider silently:

“*What would constitute a suitable delivery mode for the proposed training approach for the delivery of quality POC diagnostics services in your places of work?*”

The participants were separated into two groups of four and were provided with the necessary resources to record and present their ideas. The following steps were followed for participants to generate ideas to respond to the posed question. (i) Silent brainstorming, where participants were given 10 min to consider the question and record relevant ideas. Discussions were not allowed during this time. However, the participants could raise their hands for the attention of the facilitator if further clarity was required. (ii) Group discussions, where members were given a further 10 min to share their ideas with their group members, these were grouped into themes as they emerged and then pasted on a flip chart to be presented to the wider group. (iii) Group presentations and clarification, where each group selected one representative to present their ideas to the bigger NGT group. Presenters were probed for further clarifications and group members were encouraged to assist where necessary. The research assistant recorded all the ideas and together with the facilitator highlighted similar themes and removed duplicates. The collated results were presented to the whole group. The group agreed upon the presented ideas and approved for them to be piloted without a need for voting or ranking.

### Data Analysis

Qualitative and quantitative data was collected in this study and a mixed methods approach (18, 19) was employed to analyse data obtained. Thematic content analyses (20, 21) was used to analyse qualitative data and descriptive statistical analysis was used to analyse quantitative data. Furthermore, an analytic-algorithm built into the online course enabled monitoring of participation and assessment outcomes automatically. Activity reports were retrieved from Moodle analytics and were used to generate descriptive data on participant engagement and assessment outcomes. The use of a mixed methods approach for data analysis contributed to the trustworthiness and reliability of the research findings, enabling us to meet the aims of this study (18, 19).

### POC Diagnostics m-Learning App Development

This section reports on the development process of the mobile application (app) for the delivery of a POC diagnostics curriculum for nurses in resource-limited settings. The mobile app was identified as the preferred approach based on the NGT results and best method to present and disseminate available policies and guidelines on HIV testing, directly to participants in rural and remote settings. This section also includes the development of curriculum content and the design process of the mobile app, as well as its features and specifications.

#### Curriculum Content

According to the World Health Organization (22), basic HIV testing training for all staff should cover the following specific issues: basic HIV awareness (HIV transmission and prevention); the purpose and benefits of HIV testing and counseling; the process of HIV testing and counseling; confidentiality; how the HIV testing and counseling team works together, including roles and responsibilities and line management; health and safety, pre-test and post-test counseling; and ongoing counseling. The structure of the content provided in this study was informed by experiential learning theory (23) and Bloom's Taxonomy (24) learning framework proposed for a point-of-care diagnostics training program in recent research in the South African context (25). It was then delivered through mobile-learning as detailed in the previous study (10).

#### Technical Aspects

Mobile devices have different hardware and operating systems (OSs). Globally, smart phones tend to fall into two camps: Apple and Android. In order to take full advantage of the hardware and OS capabilities of a mobile device, developers need to create an app using an approach consistent with that device's programming environment. Apple iOS devices (iPhone, iPod Touch, iPad), use Objective-C and Apple's XCode developers' tool. However, such apps will not run on Android devices, for which apps are written in Java running on a version of Linux (26). iOS apps are only available exclusively from the Apple App Store, whereas Android apps are more widely available from different repositories including the official Android Market and Amazon's App store for Android. For the purposes of this study, the proposed mobile app was designed for Android devices, due to its affordability. The majority of South African mobile users use Android, with Android OS system market share value of 85.9% worldwide (27). Another issue to consider is that iOS development is Mac OS only, whereas app development for Android can be done on Windows or Macintosh machines, and Linux (26). Moreover, Android smart phones are more affordable and widely accessible than Apple devices in South Africa and are therefore more relevant for this study's target group.

The initially proposed app comprised of the following specifications: central panel (admin) for updating content, which includes videos and images; registration/sign-in window to keep track of new and old users; information security; data encryption; and confidentiality and privacy. The following features of the app would be made available to the users: policy documents; quality documents; video on performing quality HIV tests and images of current testing kits. Links to relevant websites including the World Health Organization (WHO), Department of Health (DoH) KwaZulu-Natal and the DoH South Africa websites were also incorporated.

As commercially designed apps are expensive, and require significant budgets to trial, an innovative approach to trialing the app content on a mobile network and test the concept was necessary. A university collaboration was set up with a private higher education institute (Stadio School of Education) to develop a pilot app (28), using an existing online learning management system (LMS) platform: Moodle. Moodle (Modular Object-Oriented Dynamic is a widely used learning platform, technically supported on a global scale and established online with a functional mobile learning app downloadable on the Google Play app store (29).

The study used an online education platform (learning environment) to create an interactive online course for participants to experience and give feedback on the content provided. Moodle is a free and open-source learning management system written in PHP and distributed under the GNU General Public License (30, 31). This platform provides for various forms of electronic learning (eLearning), which include mLearning through the Moodle app and web browser. As this was a trial learning application, for public benefit, Stadio School of Education (SSoE) gave researchers permission to use their Moodle development site to trial the educational package in real time, over the course of 4 months. They also provided technical support through their Maths, Science and Technology department and Digitally Enhanced Learning and Teaching unit (DELTA) located at their Musgrave headquarters in Durban (28, 29).

#### Participant Access to the Online Course

For ethical purposes, dummy usernames and passwords were created and loaded onto Moodle by the head of DELTA (S.G). Participants had to submit real email addresses to have their account activated but their personal ID was not loaded into the system. The participants were directed to accessing the course *via* the standard route for distance learning for accessing Moodle as follows:

1. A Uniform Resource Locator (URL) link for the Moodle app, with automatically created login credentials was sent to each participant via email.
2. Instructions for accessing the app and logging into the course were included with communications to the participants.
3. Contact sessions to orientate nurses to use the app, as well as the web browser to access the course on Moodle, were conducted.
4. Participants' involvement on the course page, and the timing of their interactions, were logged automatically using Moodle analytics. These were built into the app using a programmable feature called “activity completion.”
5. Participant activity on the course was recorded automatically and completion of the course was rewarded with “electronic badges” as motivational tool. These were also set to notify researchers of course completion.
6. At the conclusion of the course, researchers were able to access Moodle-generated activity reports which logged the participants' progress through the course, chronologically by activity.
7. Moodle reports are generated as both online visuals and downloadable Excel formats. The latter form was used to report on participation and engagement with various activities.

## Results

### NGT Results

The two nominal groups generated delivery approach ideas were categorized into five themes, namely: text, visuals, videos, audio, languages and hybrid approach, as illustrated in Table 1.

**Table 1:** Nominal group workshop generated ideas for curriculum delivery approaches.

**Delivery mode**	**Group 1**	**Group 2**
Text	-Introduction and instructions -Importance of testing and knowing your status.	-Subtitles on videos
Visuals	-Show pictures of results of not taking treatment vs. taking treatment -To encourage professional nurses to participate in HIV/AIDS testing programmes	-Visuals are important because what you have seen stays in your mind
Videos	-Show correct sitting positions -Show treating patients with respect -Show good communication skills	-Use cartoons on videos instead of human beings to avoid unnecessary stigmatisation
Audio	-A piece on the policy on the importance of confidentiality	-A piece on the policy on the importance of confidentiality
Language	All languages with english subtitles	
Hybrid	_	Mixture of cartoons, audio, and writing

Each of the delivery approaches presented by the stakeholders was relevant to the delivery of specific content. There was only one duplicate: an audio piece of the confidentiality policy; and there were no conflicting ideas.

### Developed m-Learning Application

**Figure 1:**
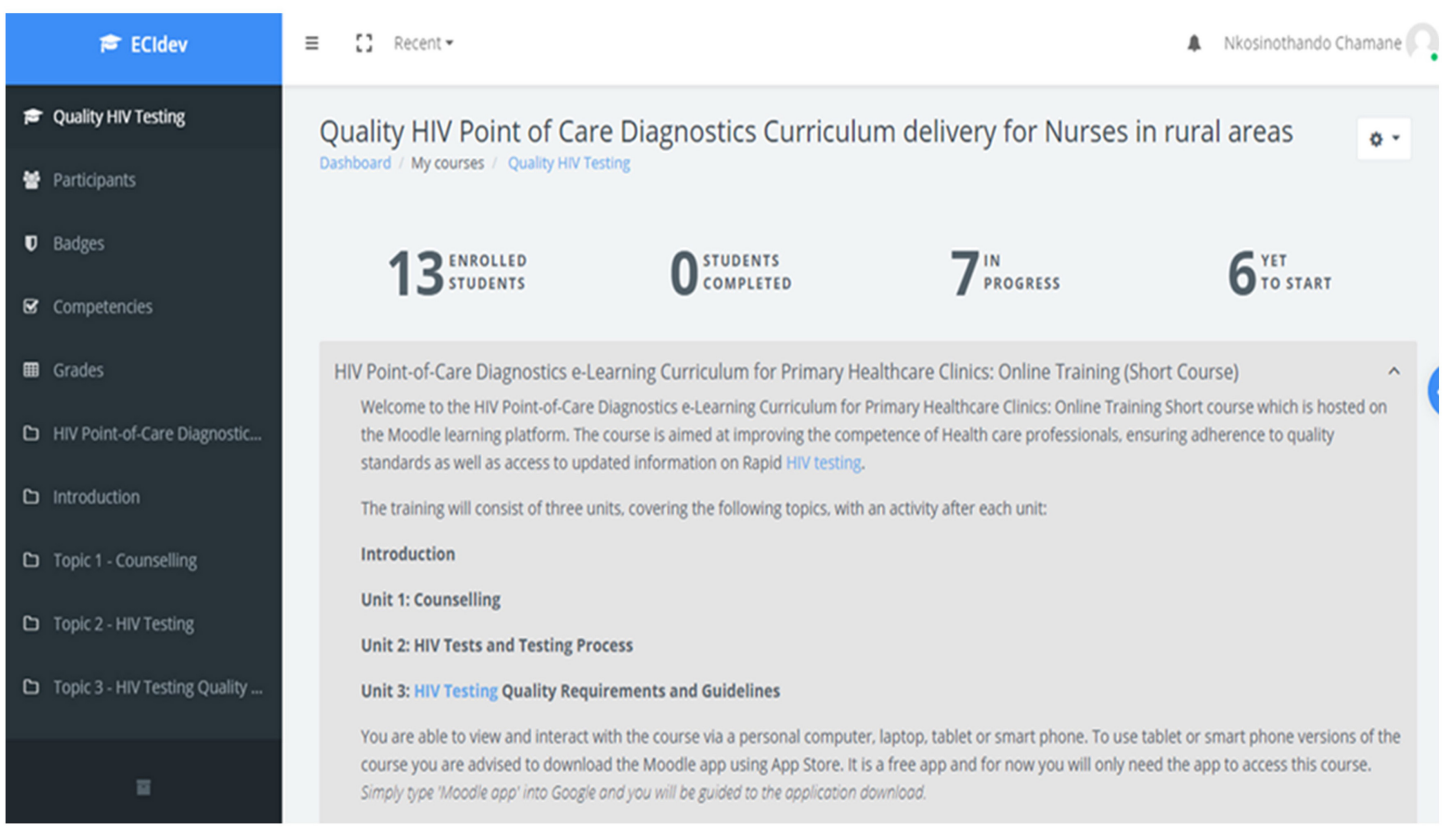
Quality HIV testing online course landing page. https://moodle-dev.embury.ac.za/login/index.php.

The interactive course created on the Moodle site was titled: Quality HIV Point-of-Care Diagnostics Curriculum Delivery for Nurses in Rural Areas. It consisted of three learning units, activities and an online quiz to assess the participants understanding, as well as achievement of competencies outlined in our previous study (10). The page also contained an online survey to evaluate the entire learning experience as elaborated further in section eLearning Course Pilot Results and Feedback. Figure 1 illustrates the dashboard of the online course.

### ELearning Course Pilot Results and Feedback

As reported in section Materials and Methodology, activity reports from Moodle analytics were used to generate descriptive data on participant engagement and assessment outcomes. From the 11 participants invited to participate on the course, 64% (*n* = 7) accessed the course. Out of the seven participants that accessed the course, 71% five (*n* = 5) were able to complete all the activities within 3 months. Course participation included: reading training content; completing various activities which included an online quiz; and a survey at the end of the course. The results of each activity completion are illustrated in Table 2.

**Table 2:** Course activity completion (*n* = 7).

**Activity**	**Completed**
1. Introduction to human immunodeficiency viruses (HIV) point-of-care diagnostics testing services	71%
2. Topic 1 – Counselling	86%
3. Forum discussion-1	71%
4. Topic 2- HIV testing	100%
5. HIV testing video	86%
6. Topic 3 - HIV testing quality requirements and guidelines	57%
7. NATIONAL HIV TESTING SERVICES: POLICY 2016	57%
8. Consolidated guidelines on HIV testing services	14%
9. Discussion Forum-2	14%
10. Online quiz	71%
11. Course evaluation survey	71%

The activity completion report results as summarized in Table 2 show that the participants prioritized the counseling and HIV testing topics, followed by the HIV testing videos and then the online quiz and survey (71–100%). Less participation was observed for topics covering Quality requirements and guidelines (14–57%).

Results from the online quiz and survey feedback, as well as reasons given for non-completion or non-participation are provided below.

#### Online Quiz Results

**Figure 2:**
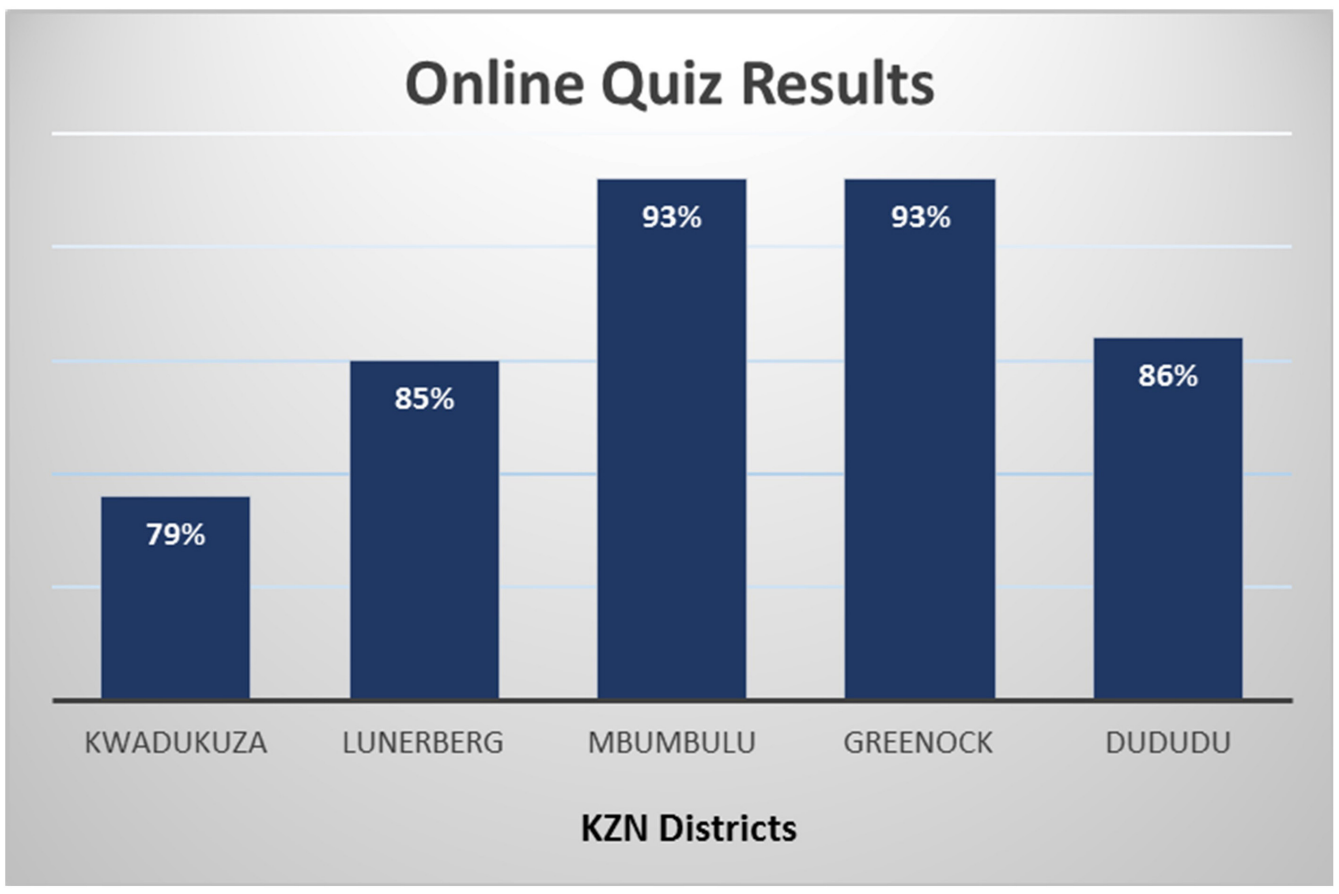
Quality HIV testing Moodle app online quiz results.

The online quiz was aimed at assessing the participants' understanding of the content knowledge as well as their ability to apply relevant quality requirements. All of the participants that attempted the quiz achieved the 75% pass mark, with a minimum result of 86%, maximum of 93% and an average of 87%. No participants scored a 100%. The distribution of participants representing each district are illustrated in Figure 2.

#### Online Survey Results

At the end of the course, each of the participants was invited to complete an online survey to critically evaluate the learning process they had experienced. This included the training content, the activities as well as the ease of access of the online course. The survey consisted of both qualitative and quantitative questions.

#### Quantitative Results

**Figure 3:**
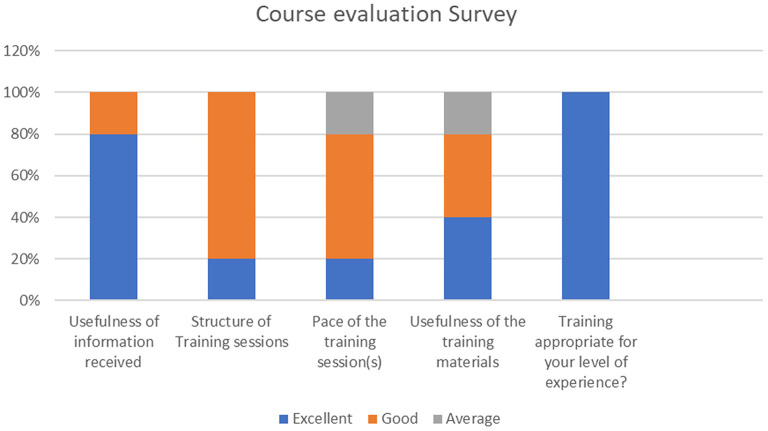
Course evaluation survey responses.

In the quantitative part of the survey, the participants had to respond by giving a score on a Likert scale from “excellent” to “poor.” All the participants deemed the training to be useful and appropriate for their level of experience. They also reported the structure of the sessions to be excellent. 60% found the pace of the training to be good, with 40% finding it not very convenient for their work schedule. 20% reported the pace and usefulness of the training material as average. The responses are illustrated in Figure 3.

#### Qualitative Results

The qualitative part of the survey gave the participants a platform to give written feedback, as well as recommend strategies to further improve the course. Guided by the structure of the survey, the following themes were identified: Theme 1: Most interesting part of the course; Theme 2: Challenges experienced; and Theme 3: Recommendations toward continuous improvement. The findings are presented below:

**Theme 1: Most interesting part of the course**

Participants reported that they enjoyed the video segment of the course as well as the fact that they could do the course at their own pace. Others reported that the content was like a refresher course for them, and they also learnt new things about the different testing kits available. A professional nurse who had just been re-introduced back to HIV Testing in a couple of years, had this to say about the online experience via a mobile app:

“*That training was a refresher course, provided on- line, one can actually download documents & guidelines through smartphone and refer to during patient's consultation.”*

**Theme 2: Challenges experienced**

One participant reported that they did not experience any challenges since the content covered was made up of the work they performed on a daily basis. The majority reported network problems since the links required them to go online and use data. Furthermore, they reported that it took them longer to complete the course when using their personal phones than when using a computer. This was more of a common challenge due to shared workstations. The participants also complained about having to participate in a quiz, one identifying some missing information in one of the questions.

Former HIV/AIDS counselor: “*Question 1 of the quiz was not done well, because there was no question, it was answers only*.”

This was confirmed and rectified, but only after the course had already been closed to participants.

Another participant had this to say about the quiz:

Professional Nurse: “*I was not comfortable doing the quiz, concerned I might fail and feel incompetent.”*

The participant went on to say that she preferred the written activities more, as she could elaborate on her responses.

**Theme 3: Recommendations toward continuous improvement**

The participants gave direct recommendations as follows:

- PAR-1: “*Present the whole course as a video.”*
- PAR-2: “*Make the course be available in native languages, depending on the target areas preferred language.”*
- PAR-3: “*Make a video of how to download the Moodle App.”*
- PAR-4: “*Include a practical activity.”*

The participants recommended that the entire course be presented as a full instructional video and be available in other South African languages, as well as include a practical activity.

### Reasons for Non-participation or Non-completion of the Course

The participants who did not manage to access the course, and those who were able to access the course, but did not manage to complete all the activities, reported the following challenges: poor network coverage in their area, unavailability of resources to access the internet (smart phones, computers, and data) and time constraints due to reprioritization of duties to deal with the Covid-19 pandemic.

Two participants reported that they felt they were too old for online courses and requested that the PI revisit their clinics to provide the training in a traditional way, with presentations, group discussions and hand-written tests.

## Discussion

The aim of this study to use stakeholder generated delivery approaches to design and pilot an evidence-based, and context specific curriculum to improve the quality of POC diagnostics services in rural clinics of KZN, was met. We observed a high participation rate and moderate completion rate during the piloting stage of the project. The group average mark of 87% for the online quiz demonstrates that the content was presented at a fair level of understanding for participants, with minor challenges. Though the quiz was shown to be a useful tool for online assessment, one of the participants expressed an apprehension concerning how their performance would be viewed. Though this aspect was address through the use of dummy usernames to protect participant identity, it is important to take this issue into consideration and look at the possibility of using other assessment tools when designing the course for a scaled-up intervention.

Quantitative and qualitative data was also generated from the online survey, where the participants provided feedback on their experiences with the course content and access. All the participants gave positive feedback (above average ratings) on the delivery of the course content. The findings of this study are in harmony with those of other studies conducted in similar settings. Similar to Pimmer et al.'s 2014 study, participants also gave positive feedback, highlighting that the tools provided through mLearning were shown to be suitable to link health workers in marginalized areas to more opportunities of learning (3). In another study, nursing students reported numerous benefits of using mobile technology, such as reduced levels of anxiety due to learning in practice, better access to educational material, knowledge improvement and confidence (32). However, access for participants in our study was a challenge to some of the participants hence the 64% participation rate. The qualitative responses were categorized according to three themes: (1) Most interesting part of the course, (2) Challenges experienced, and (3) Recommendations toward continuous improvement. The video segment on the course, the provision that participants could do the course at their own pace and the ease of availability of up to date information via mobile technology were reported amongst the most interesting parts of the course. However, access to network, technology resources, and downloading of attached links and documents were reported as the main challenges to accessing and completing of the course.

The barriers to access to mLearning identified in this study also resonate with those reported in other research studies in Africa. In a study on the adoption of mLeaning in clinical nursing education, barriers such as the negative attitudes of nursing staff toward trying new programs and poor Wi-Fi connectivity were also identified as some of the issues preventing full adoption of mLearning (O'Connor and Andrews, 2018). These issues, and others related to access to telecommunications networks and high data cost, are common to most students studying at a distance in South Africa, considering that this country had been identified as one of the nations with the most inflated data costs (33), further worsened by the challenges in the era of Covid-19 (34).

### Summary of Findings

In the first part of this study, the relevant stakeholders participated in an NGT workshop, where five delivery modes for the curriculum content through mLearning were recommended: text, audio, images, videos, and a combination of all the presented delivery modes. Each of the delivery modes presented was relevant to the delivery of a specific part of the content, and all participating stakeholders agreed to the design without objections. They also recommended that content be presented using multiple languages. However, this idea could not be incorporated due to cost limitations for translation services. This was also recommended by participants at piloting of the mLearning platform, emphasizing that presenting content in multiple languages would accommodate and attract more users. An online activity completion report was extracted and the results showed that other topics were accessed more than others. Counseling and HIV testing topics, followed by the HIV testing video were accessed the most, with less participation observed for topics covering quality requirements and guidelines. The topics prioritized were presented through images, videos and simple text. Less prioritized topics were those presented as attachments of national documents. The drop in participation on these topics may be attributed to challenges associated with reading of large documents, as well as access and connectivity issues. In related research, mLearning was found to be mainly applied to the training of basic nursing concepts and to long-term care, with a few published studies related to other nursing education domains such as POC diagnostics (1). Furthermore, there is a need for the development of mLearning policies to explicitly address and consider the intrinsic economic, social and regional inequalities existing within African countries (35).

### Study Limitations and Strengths

Due to financial constraints, the development of a stand-alone, locally developed and sponsored app to be installed on users' devices could not be developed. However, findings of this study will guide the next phase recommended for implementation. The provision of course content in multiple languages as well as subsidizing of willing participants with resources and data to boost the participation rate was also not possible. However, Android applications allow users to utilize translation tools provided by search engines and video platforms such as Google and YouTube. YouTube has a feature which provides translation services to videos saved on their channels. A low-cost application may be more ideal in assisting with translations, however, with eleven official languages, South Africa faces severe challenges with translating learning materials for all home languages.

This study demonstrated that utilizing open access platforms such as Moodle, through private higher education institutions (HEIs) can be mutually beneficial. This study collaborated with information technology and pedagogical experts at Embury Institute of Higher Education without having to commission commercial development of an untested app.

### Recommendations

Based on the positive reception with challenges on the uptake of a POC diagnostics curriculum for quality HIV/AIDS testing using mobile technology, the following are recommended for future implementation: Collaboration with specialists in eHealth to use the findings of this study to develop a national health service app with searchable sections including all other POC tests listed on the essential diagnostics list. Collaboration or dialogue with cell phone networks to establish subsidized or free data for essential health workers. Investment on the provision of reliable technology resources and training of health care workers on using smart phone technology to optimize data usage as well as on how to learn in offline and online environments. Furthermore, we recommend a presentation or delivery of quality guidelines using new approaches, which include videos and power point presentations.

## Conclusion

A POC diagnostics curriculum for quality HIV/AIDS testing through mLearning was designed and well-received due to its numerous benefits for PHC clinics in rural areas of KZN. However, barriers due to poor network coverage and lack of technology resources still need to be addressed to better prepare for the vast advancements in the provision of training as well as other health care services through the use of mobile technology. Collaborations between specialists in eHealth and network providers to establish subsidized applications for health workers in remote areas are recommended.

## Data Availability Statement

The original contributions presented in the study are included in the article/supplementary material, further inquiries can be directed to the corresponding author/s.

## Ethics Statement

The studies involving human participants were reviewed and approved by KwaZulu Natal Health Research Committee (KZ_201904_008) and the University of KwaZulu-Natal (UKZN) Biomedical Research Ethics Committee (BF514/18). The patients/participants provided their written informed consent to participate in this study.

## Author Contributions

NC and TM-T conceptualized the study. The training content was conceptualized by NC and edited by TM-T, verified and loaded onto Moodle by NC following training at Stadio School of Education. RT provided Moodle training and managed provision of participation and processing of course analytics. SG provided gatekeeper permission for use of the site, created login credentials, and loaded all participants onto the Moodle course. The study was continuously updated and verified by all collaborators as more information became available. All authors contributed to the article and approved the submitted version.

## Conflict of Interest

The authors declare that the research was conducted in the absence of any commercial or financial relationships that could be construed as a potential conflict of interest.

## Publisher's Note

All claims expressed in this article are solely those of the authors and do not necessarily represent those of their affiliated organizations, or those of the publisher, the editors and the reviewers. Any product that may be evaluated in this article, or claim that may be made by its manufacturer, is not guaranteed or endorsed by the publisher.
